# Prenatal Diagnosis of Umbilical Cord Ulcer: A Report of Two Cases

**DOI:** 10.1155/2019/3768761

**Published:** 2019-12-19

**Authors:** Daisuke Katsura, Suzuko Moritani, Shunichiro Tsuji, Kaori Hayashi, Kazutaka Yamada, Shinsuke Tokoro, Kounosuke Suzuki, Fuminori Kimura, Takashi Murakami

**Affiliations:** ^1^Department of Obstetrics and Gynecology, Shiga University of Medical Science Hospital, Setatsukinowa-cho, Otsu, Shiga 520-2192, Japan; ^2^Department of Diagnostic Pathology, Shiga University of Medical Science Hospital, Setatsukinowa-cho, Otsu, Shiga 520-2192, Japan

## Abstract

Umbilical cord ulcer is related to fetal intestinal atresia or meconium; perforation of the ulcer causes fetal deterioration leading to fetal and neonatal death owing to fetal hemorrhage. However, to the best of our knowledge, a method to diagnose umbilical cord ulcer prenatally is not available. No reports exist about the prenatal findings before perforation of umbilical cord ulcer using ultrasonography. We encountered two cases of umbilical cord ulcer showing ultrasonographic finding of a linear echo around the umbilical cord. Umbilical cord ulcers with an exposed umbilical cord artery in the first case and with perforation of the artery in the second case were diagnosed postnatally. When we encounter such ultrasonographic finding, especially with polyhydramnios and high amniotic bile acid concentration in cases of fetal intestinal atresia, risk of perforation of the umbilical cord ulcer should be included in the differential diagnosis.

## 1. Introduction

Umbilical cord ulcer (UCU) was first reported by Blanc and Allan [1]. It is related to fetal intestinal atresia and occurs in 5.6% of fetuses with intestinal atresia [2]. Congenital intestinal atresia, with an estimated incidence of approximately 1 in 5000 live births, shows ultrasonographic findings of gastrointestinal dilatation such as a double, triple, or multiple bubble sign [3]. The perinatal mortality rate in fetuses with intestinal atresia was approximately 10% [4]. In addition, UCU has been reported to be caused by meconium [5]. The mortality rate in fetuses with UCU was approximately 46% [6]. As UCU perforation causes acute fetal blood loss leading to fetal death, an emergency delivery is often required [1, 2]. However, as the fetal condition deteriorates rapidly after perforation, delivery before perforation is desirable. The degree of UCU is graded based on its width: from the desquamation of the epithelium (grade 1) and detachment of the basal lamina (grade 2) to the thinning of Wharton's jelly with widespread grade 2 changes (grade 3) and exposed umbilical artery or vein (grade 4), in addition to perforation [7]. Therefore, it may be valuable to diagnose UCU and determine the risk of perforation prenatally. Ultrasonographic and amniotic findings for such diagnoses have been discussed; however, they have not been established yet. Herein, we present our experience of two interesting cases with the same ultrasonographic findings: one case of UCU grade 4 and one case of UCU perforation.

## 2. Case Presentation

### 2.1. Case 1

A 31-year-old woman with polyhydramnios at 31 weeks of gestation was admitted to our hospital owing to threatened premature labor. Fetal ultrasound examination revealed that the maximum vertical pocket (MVP) was 8.0 cm, amniotic fluid index (AFI) was 30 cm, and estimated fetal weight (EFW) was 1556 g (26.4 gestational age percentile). The double bubble sign was observed in the fetal abdomen, and fetal duodenal atresia was diagnosed. Because the patient had frequent uterine contractions and cervical dilatation, amnioreduction was performed at 31 weeks and at 32 weeks of gestation. During the first amniocentesis, we performed amniotic diagnosis for karyotyping and made a diagnosis of trisomy 21. The patient was provided information about the genetics and clinical course of trisomy 21. The amniotic bile acid concentration was 16.6 *μ*mol/L and 16.5 *μ*mol/L during the first and second amniocenteses, respectively. Thereafter, the patient's condition was stable.

**Figure 1:**
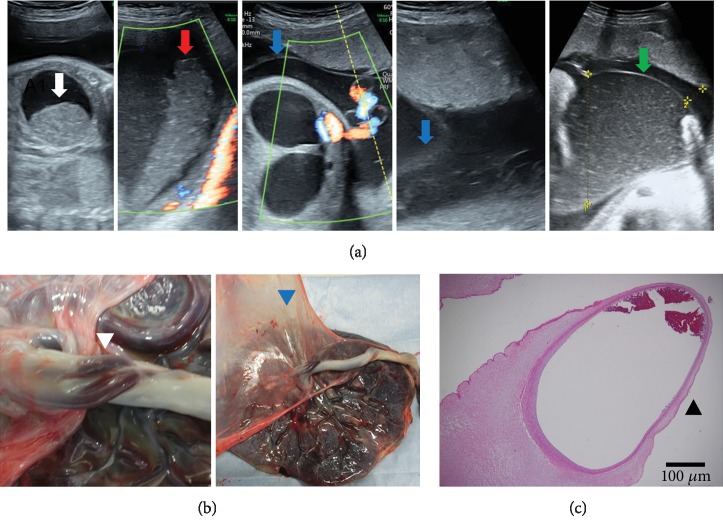
Case 1. (a) Ultrasonographic findings at 36 weeks of gestation. Heterogeneous high echoic mass-like debris within the fetal stomach (white arrow) and uterus (red arrow). Linear echo findings around the umbilical cord (blue arrow) and chorioamniotic membrane separation (green arrow). (b) Macroscopic findings. Umbilical ulcer with exposed umbilical cord artery (white arrowhead). The amniotic and chorionic membranes were separated on the surface of the placenta (blue arrowhead). (c) Original magnification. Loss of the amniotic membrane, degeneration of the umbilical cord stroma, and exposure of the umbilical cord artery (black arrowhead, hematoxylin and eosin; H&E, ×40).

At 36 weeks of gestation, fetal ultrasound findings showed heterogeneous high echoic mass-like debris within the uterus and fetal stomach, linear echo around the umbilical cord, and chorioamniotic membrane separation (Figure 1(a)). Because these findings were not confirmed 5 days earlier, fetal heart rate was fully monitored on the cardiotocogram. The umbilical artery pulsatility index (UmA PI), middle cerebral artery (MCA) PI, and MCA peak systolic velocity (PSV) were 1.33, 2.14, and 79.75 cm/s (1.49 MoM), respectively, in the fetal Doppler. Although the fetal heart rate pattern remained reassuring on cardiotocogram, we could not deny the risk of UCU perforation considering these findings and performed a cesarean section around 12 hours later. The amniotic fluid was not blood tinged.

A female infant weighing 2282 g with Apgar scores of 7 and 9 at 1 and 5 min, respectively, umbilical artery pH of 7.352, and hemoglobin concentration (Hb) of 13.0 g/dL was delivered. After birth, a large amount of bile-like yellowish brown fluid was aspirated from the infant's stomach, and her respiratory and circulatory states were stable. She had mild anemia but did not require any treatment. Although fetal hemoglobin and alpha-fetoprotein levels were not measured, fetomaternal transfusion was suggested as the cause of anemia because there was no bleeding, no blood group incompatibility, and no other apparent cause. The infant underwent an operation for duodenal atresia at 2 days after birth. Duodenal atresia type IIIa, distal from the ampulla of Vater, was diagnosed. The postoperative course was uneventful and the infant was discharged at age 39 days.

Macroscopically, two UCUs with exposed umbilical artery were observed at the umbilical cord surface attached to the placenta (Figure 1(b)). The amniotic and chorionic membranes were separated from the surface of the placenta (Figure 1(b)). Histopathological examination revealed amniotic membrane loss, umbilical cord stroma degeneration, and umbilical artery exposure (Figure 1(c)).

### 2.2. Case 2

A 38-year-old woman with polyhydramnios at 30 weeks of gestation was admitted to our hospital owing to threatened premature labor. On fetal ultrasound examination, the MVP, AFI, and EFW were 12.2 cm, 31.5 cm, and 1388 g (26.3, gestational age percentile), respectively. The double bubble sign was observed in the fetal abdomen, and fetal duodenal atresia was diagnosed. Because the patient had abdominal distension and frequent uterine contractions, amnioreduction was performed. At this point, the amniotic bile acid concentration was 25.3 *μ*mol/L. Thereafter, her condition was stable.

**Figure 2:**
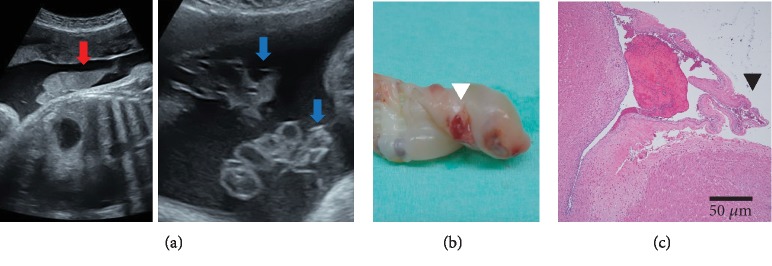
Case 2. (a) Ultrasonographic findings at 36 weeks of gestation. Heterogeneous high echoic mass-like debris within the uterus (red arrow) and linear echo finding around the umbilical cord (blue arrow). (b) Macroscopic findings. Umbilical ulcer with perforation of umbilical cord artery (white arrowhead). (c) Original magnification. Focal perforation of the umbilical cord artery with thinning of Wharton's jelly (black arrowhead, H&E, ×100).

At 34 weeks of gestation, it was difficult to confirm the fetal heart rate on cardiotocogram. On fetal ultrasound examination, heterogeneous high echoic mass-like debris within the uterus and linear echo findings around the umbilical cord were detected (Figure 2(a)). These findings were not confirmed 2 days earlier. Fetal myocardium movement seemed weak, UmA PI of 0.97, MCA reversed end-diastolic velocity, and MCA PSV of 25 cm/s were seen, and the fetal heart rate was 80-110 bpm as observed by fetal Doppler. We speculated fetal circulatory insufficiency due to fetal hemorrhage caused by UCU perforation; we performed an emergency cesarean section. The amniotic fluid was blood tinged. After surgery, from the images, the calculated left and right ventricle myocardial performance index was 0.67 and 0.70, respectively, and the left and right ventricle fractional shortening was 10% and 9%, respectively.

A female infant weighing 2086 g was delivered; the infant required resuscitation with intubation, heart massage, and epinephrine. Her Apgar scores were 1, 3, 3, and 5 at 1, 5, 10, and 15 min, respectively. Her umbilical artery pH was 7.157, base excess was -9.6, and Hb was 7.9 g/dL. Results of the neonatal laboratory tests were as follows: Hb, 6.9 g/dL; platelets at birth and one day later, 12.8 × 10^4^ *μ*L and 3.1 × 10^4^ *μ*L, respectively; fibrinogen, 30 mg/dL; prothrombin time—international normalized ratio, 6.29; and D-dimer, 21.8 *μ*g/mL. Neonatal anemia and disseminated intravascular coagulation were observed. The infant received repeated blood transfusions and was placed on conventional mechanical ventilation. The Thompson score was 12, and the case was classified as severe encephalopathy. Brain diffusion-weighted magnetic resonance imaging showed diffuse hyperintensity throughout the cerebral cortex, which suggested diffuse hypoxic-ischemic necrosis and hypoxic ischemic encephalopathy. Chromosomal abnormality was not confirmed.

Macroscopically, one UCU with umbilical artery perforations and one UCU with exposed umbilical artery were observed at the umbilical cord surface attached to the placenta and fetus (Figure 2(b)). Histopathological examination revealed focal perforation of the umbilical cord artery with thinning of Wharton's jelly (Figure 2(c)).

## 3. Discussion

Prenatal diagnosis of UCU and assessment of the risk of perforation of UCU using ultrasonography are yet to be established. To our knowledge, these are the first report of UCU cases with ultrasonographic findings that were suggestive of a risk of UCU perforation. The finding of linear echo around the umbilical cord might be helpful as a marker to predict UCU perforation.

Although the causes of UCU are unclear, congenital abnormalities of the umbilical cord epithelium, ischemia caused by abnormal spasm of the umbilical vessels, and degeneration caused by digestive enzymes in fetal vomiting [8] may be potential causes. These factors alone or in combination might cause UCUs [8]. Congenital abnormalities of the umbilical cord epithelium might be related to chromosomal anomalies, structural anomalies, fetal growth restriction, abnormal insertion of the umbilical cord, and umbilical cord abnormalities, such as noncoiled umbilical cord, umbilical cord cysts, umbilical vein varices, and single umbilical artery [9]. In a previous report, bile toxicity was suggested as the cause of UCU based on pathological findings that revealed activated macrophages containing pigment granules within the ulcer bed, necrotic areas of Wharton's jelly, and fetal membranes [10]. Not only digestive enzymes but also meconium reportedly cause ischemia and degeneration of umbilical vessels, leading to the development of UCU [5].

Regarding the heterogeneous high echoic mass seen on ultrasound, amniotic fluid sludge indicating the presence of bacteria and intra-amniotic inflammation could be responsible for it [11]. However, intra-amniotic inflammation was not found in both our cases. Therefore, we speculated that the heterogeneous high echoic mass-like debris within the fetal stomach and uterus indicated a high-density mass, including digestive enzymes, such as bile acid, caused by intestinal atresia as in case 1, in which nonbloody amniotic fluid and bile-like fluid were aspirated from the stomach; there was linear echo finding around the umbilical cord, and chorioamniotic membrane separation indicated exposure to highly concentrated bile acid. However, the debris within the uterus in case 2 might indicate blood clot because of bloody amniotic fluid. Considering the ultrasonographic findings of UCU, spontaneous bleeding from the umbilical cord [12] and a hazy substance flowing near the fetal navel in the amniotic cavity [13] have been reported. In these cases, the UCU had ruptured already. Although our cases showed similar ultrasonographic findings, case 1 was of UCU with exposed umbilical cord artery and case 2 was of UCU with perforation of the umbilical cord artery based on histopathological findings.

In cases of fetal intestinal atresia, although bile acid concentration in the amniotic fluid has been found to be high, the relationship between bile acid concentration and UCU remains unclear [14, 15]. However, Nakamura et al. reported that UCU was associated with high bile acid concentration in the amniotic fluid [4]. They showed that UCU was detected in more than 90% of cases with bile acid concentrations ≥ 10 *μ*mol/L in the amniotic fluid [4]. In intestinal atresia with polyhydramnios, we measure bile acid concentration when amnioreduction. If the bile acid concentration is ≥10 *μ*mol/L, hospitalization continues, cardiotocogram is performed twice daily for 40 minutes, and ultrasonography is performed twice a week. In our cases, the amniotic bile acid concentration was ≥10 *μ*mol/L. In addition, UCU perforation was reported after 26 weeks of gestation [2, 4, 10], which covers the time (30.0 ± 3.5 (25-7) weeks of gestation) of polyhydramnios development in fetal intestinal atresia [16]. Polyhydramnios might suggest that the umbilical cord is exposed to a high concentration of digestive enzymes, which may be considered as a risk factor for UCU.

Regarding common sites of UCU, there are various reports of UCU occurring on the fetal side [17], on the placental side [5], or as a line or spot along umbilical artery [6]. UCUs were confirmed in the placental side in case 1 and in the fetal and placental side in case 2. The location of UCU might be related to the degree of exposure to digestive enzymes and may depend on the location of the placenta, umbilical cord, and fetus.

We have carefully incorporated ultrasound findings after managing the present two cases; subsequently, we experienced four cases of fetal duodenal atresia. All cases had amnioreduction, and the bile acid concentration was ≥10 *μ*mol/L except in one case. One case with high bile acid concentration had UCU grade 2, and the other cases had no UCU. None of these cases had chromosomal anomalies and any unusual findings including the ultrasonographic findings. Therefore, our ultrasonographic finding might reflect perforation and UCU grade 4. Although UCU grade 4 was associated with intrauterine fetal death [4], we could save the fetus, and it appeared that there was a time delay between confirming these findings and UCU perforation. However, the degree to which these findings represent the risk of UCU perforation could be the subject of a future study. In addition, although we detected these ultrasonographic finding of linear echo around the umbilical cord and chorioamniotic membrane separation, these are insufficient for an accurate diagnosis of UCU. Amnioreduction might cause these ultrasonographic findings because chorioamniotic membrane separation is induced by medical intervention, such as amniocentesis and fetal surgery [18]. Chorioamniotic membrane separation has been reportedly related to chromosome abnormalities [19]. Therefore, further data are needed to clarify the efficacy of these ultrasonographic findings, and it is necessary to comprehensively evaluate these ultrasonographic findings. Careful judgment considering gestational age is required for delivery because premature delivery increases neonatal morbidity.

In summary, in case of such ultrasonographic findings, especially with polyhydramnios and high amniotic bile acid concentration in cases of fetal intestinal atresia, the risk of UCU perforation should be considered.
